# Full‐Color Emission Polymer Carbon Dots with Quench‐Resistant Solid‐State Fluorescence

**DOI:** 10.1002/advs.201700395

**Published:** 2017-09-28

**Authors:** Jieren Shao, Shoujun Zhu, Huiwen Liu, Yubin Song, Songyuan Tao, Bai Yang

**Affiliations:** ^1^ State Key Laboratory of Supramolecular Structure and Materials College of Chemistry Jilin University Changchun 130012 P. R. China; ^2^ Department of Chemistry Stanford University Stanford CA 94305 USA

**Keywords:** full‐color emission, multicolor nanocomposite, polymer carbon dots, quench‐resistant emission, sub‐fluorophore

## Abstract

Polymer carbon dots (PCDs) represent a new class of carbon dots (CDs) possessing sub‐fluorophores and unique polymer‐like structures. However, like small molecule dyes and traditional CDs, PCDs often suffer from self‐quenching effect in solid state, limiting their potential applications. Moreover, it is hard to prepare PCDs that have the same chemical structure, exhibiting full‐color emission under one fixed excitation wavelength by only modulating the concentration of the PCDs. Herein, self‐quenching‐resistant solid‐state fluorescent polymer carbon dots (SSFPCDs) are prepared, which exhibit strong red SSF without any other additional solid matrices, while having a large production yield (≈89%) and a considerable quantum yield of 8.50%. When dispersed in water or solid matrices in gradient concentrations, they can exhibit yellow, green, and blue fluorescence, realizing the first SSFPCDs with the same chemical structure emitting in full‐color range by changing the ratio of SSFPCDs to the solid matrices.

Great increasing types of fluorescent materials opened up many exciting new avenues of bioimaging, sensor, and nanocomposite, yet most of fluorescent materials made from metal and small molecules suffer from the functionality and toxicity concerns.^1^, ^2^ Polymer carbon dots (PCDs), a new type of fluorescent carbon dots (CDs), prepared through polymerization and crosslinking between small molecules, have emerged and attracted increasing interest due to their unique structures and excellent properties including low toxicity, excitation‐dependent luminescence, low cost, chemical inertness, and excellent biocompatibility.^3^, ^4^, ^5^, ^6^, ^7^, ^8^, ^9^, ^10^, ^11^, ^12^, ^13^, ^14^, ^15^, ^16^, ^17^, ^18^, ^19^ The “core–shell” structure of PCDs distinguishes them from other small molecules and carbon based fluorophores by small molecule/polymer crosslinking into the emission center and holding outer polymer chains synchronously.^3^, ^4^, ^6^ Instead of containing typical conjugated chromophore, whose band gap information is affected by heteroatom doping and finite size effects,^20^, ^21^ the PCDs only possess sub‐fluorophores, a potential fluorophore (which are nonconjugated groups such as heteroatom‐containing double bonds like C=N, C=O, N=O and single bonds like amino based groups, C—O), with intrinsically very weak photoluminescence (PL). Fortunately, the fluorescence of these sub‐fluorophores in PCDs can be enhanced through physical immobilization or chemical crosslinking of polymer chains, which is entitled to the crosslink enhanced emission (CEE) effect.^5^, ^7^, ^22^, ^23^ During CEE process, the rotation and vibration of these sub‐fluorophores are efficiently immobilized, leading to an enhanced radiative transition. For example, Dai and co‐workers investigated PCDs from ethylenediamine and carbon tetrachloride (CTC), which is formed through polycondensation.^12^ Yu and co‐workers reported fluorescent nanoparticles from crosslinked phenol formaldehyde resin.^14^, ^15^ Yang and co‐workers have utilized branched polyethyleneimine (PEI) as a model system. The non‐conjugated polymer dots (NCPDs) were prepared by CTC crosslinking.^7^ Although the initial PEI possessed very weak fluorescence, the NCPDs, after CTC crosslinking, possessed elevated PL. In these four cases, immobilized by the crosslinked polymer structure, the potential sub‐fluorophores (amino and phenol) exhibited increased PL properties.

However, one of the major drawbacks of PCDs is aggregation‐caused quenching in solid/powder state, which is also observed in small molecular fluorophores, preventing all direct applications from utilizing the remaining solid state fluorescence in optoelectronic devices and sensors.^24^, ^25^, ^26^ To circumvent this drawback, encapsulation methods to immobilize PCDs in solid matrix, such as starch,^27^, ^28^ inorganic salt,^29^ silica gel,^30^, ^31^ oligomeric silsesquioxane,^32^ and polymers^33^, ^34^, ^35^, ^36^ have been adopted to disperse the PCDs in these solid matrixes to retain the fluorescence. In detail, Qu and co‐workers used inorganic salt (BaSO_4_) to stabilize the PL properties of PCDs, preventing PCDs from self‐quenching effect in solid state.^29^ PCDs, encapsulated by polyhedral oligomeric silsesquioxane were also achieved with high quantum yield (QY).^31^ Although with these treatments people have achieved solid state fluorescence (SSF) of PCDs powder, the used blending/hybrid route always has aggregation or instability issues. Nearly no reported work has successfully conquered the aggregation‐caused quenching drawbacks by developing efficient PCDs with newly designed structure and emission center.

Besides, all prior works on PCDs are solely related to blue or green emission due to the large band gap of the immobilized sub‐fluorophores, the main fluorescence centers of PCDs, limiting their promising practical applications.^5^, ^37^ It is very important and attractive to prepare PCDs with full‐color emissions from blue to red. Although some reports achieved multicolor emission by synthesizing different types of PCDs with tunable PL centers, the as‐prepared PCDs were not the same type of materials and needed change excitation wavelength to obtain full‐color emission.^38^, ^39^, ^40^, ^41^, ^42^, ^43^ As a result, it is still difficult to realize PCDs that have the same chemical structure and meanwhile exhibit full‐color emission under one fixed excitation wavelength.

To solve the aforementioned problems associated with obtaining full‐color emission PCDs, and to achieve longer fluorescence in the solid state, herein, we prepared full‐color PCDs showing concentration controlled emission with the QY of 18.9%, 8.44%, 5.54%, and 8.5% in accordance with the red shift of emission wavelength. The PCDs possess self‐quenching‐resistant SSF, exhibiting strong red SSF without any other solid matrices, while have a considerably large production yield (≈89%) and a QY of 8.50%. These new PCDs allowed us to achieve red, yellow, green, and blue fluorescence when dispersed in water or solid matrices with tunable concentrations, demonstrating the first report for SFFPCDs without any other solid matrices emitting in the red light region. Moreover we also realized the first wide tunable SSF under UV excitation with developing many interesting and novel applications in the fields of bulk fluorescent materials, multicolor inks as well as full/white color light‐emitting diodes (LEDs).

The PCDs were prepared through a microwave‐assisted method, which was described in the Experimental Section. The formation of SSFPCDs was conducted first by condensing process and 1,4 Michael reaction between maleic acid (MA) and ethylenediamine (EDA) to form the polymer‐like structure, which then crosslinked to form the SSFPCDs, maintaining the inner polymer core with net structure (**Scheme** **1**). The morphologies of SSFPCDs were characterized using transmission electron microscopy (TEM). Drops of SSFPCDs aqueous solution with the concentration of 1 mg mL^−1^ were deposited on carbon‐coated copper grids for TEM. The morphology characterization illustrated that SSFPCDs were well dispersed and exhibited spherical particle shape. Besides, no carbon lattices were observed in these particles, indicating that no graphite structure formed during the microwave process.^44^ The average size of SSFPCDs was 15 nm with a wide distribution from 8 to 22 nm (**Figure** **1**a). From the dynamic light scattering (DLS) analysis, the average particle size in aqueous state shows an average particle size of 51 nm, four times than that in dry state, which is due to the polymer structure in PCDs. Since those polymer structures in PCDs tend to absorb water, the PCDs will likely to have an extended size in aqueous state. The sizes of SSFPCDs were also measured by atomic force microscopy (AFM) and the average height was 2.50 nm (Figure 1b). The X‐ray powder diffraction (XRD) patterns of SSFPCDs showed a strong diffraction centered at 4.9 Å, which was also attributed to polymer structure or some amorphous carbon forms (Figure S1a, Supporting Information).^33^

**Scheme 1:**
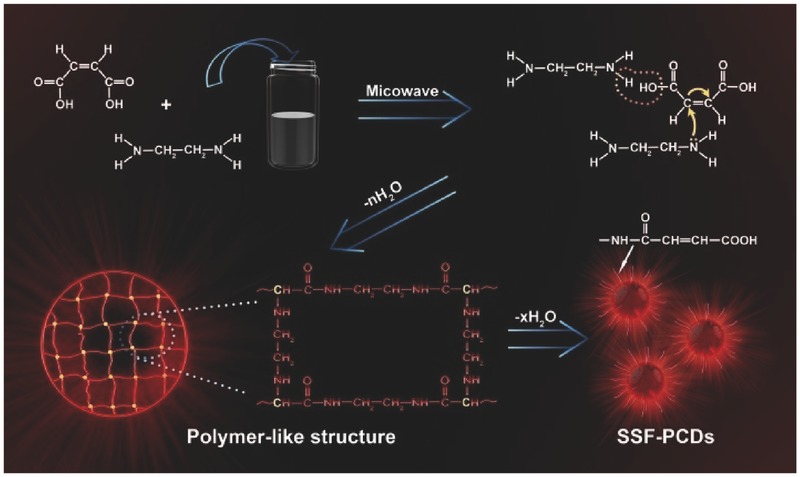
A synthetic route using maleic acid and ethylenediamine to form PCDs with possible polymer‐like structure: from condensation, crosslinking, polymerization, and partly carbonization. The concentration‐dependent fluorescence of PCDs was utilized to many of novel applications.

**Figure 1:**
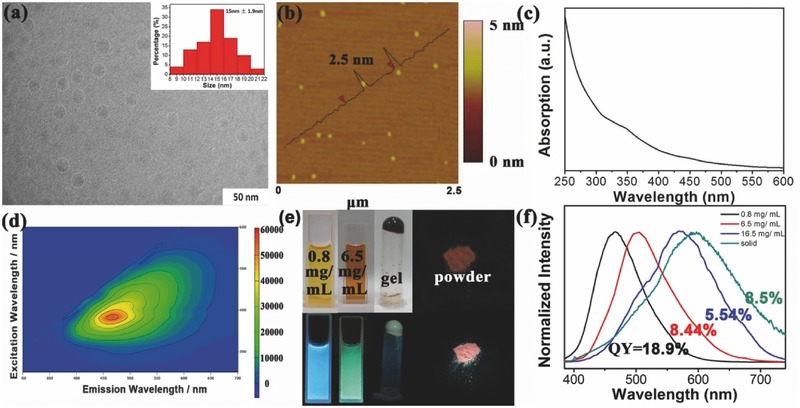
Morphology and optical properties of PCDs. a) TEM image, b) AFM image, and c) UV/Vis absorption spectra of SSFPCDs. d) Excitation–emission matrix of SSFPCDs in aqueous solutions with the concentration of 0.8 mg mL^−1^. e) Photographs of the SSFPCDs in aqueous solution with different concentrations and the SSFPCDs powder under UV light. f) The normalized PL spectra of SSFPCDs solution with different concentrations and the SSFPCDs powder under the excitation wavelength of 365 nm. Insets: a) the size distributions of SSFPCDs. b) the height of most of the SSFPCDs is 2.5 nm. f) The inset numbers are their quantum yields.

The chemical compositions of SSFPCDs were also investigated. X‐ray photoelectron spectroscopy (XPS) of SSFPCDs showed three strong peaks at 284.0, 400.0, and 530.6 eV (Figure S1b, Supporting Information), which were ascribed to C 1s, N 1s, and O 1s, respectively. In the high‐resolution spectra, the C 1s spectrum of SSFPCDs showed three peaks at 284.5, 285.5, and 288.0 eV (Figure S1c, Supporting Information) which were attributed to sp^2^ C—C, C—N/C—O, and C=N/C=O, respectively.^12^ The N 1s spectrum of NPDs showed two peaks at 399.7 and 400.6 eV (Figure S1d, Supporting Information), which were ascribed to the pyridinic N and pyrrolic N, respectively, indicating the existence of both dopant‐N atoms (C—N—C, N—(C)_3_) and amide—N (N—H).^45^, ^46^ And investigated by energy dispersive X‐ray spectrometer, the atomic ratio of carbon, nitrogen, and oxygen was 48.25%, 18.48%, and 26.99%, respectively (Table S1, Supporting Information). The surface functional groups of SSFPCDs were investigated by Fourier transform infrared (FT‐IR) (Figure S1e, Supporting Information). In the FT‐IR spectrum of SSFPCDs, the following peaks were observed: stretching vibrations of C—OH at 3430 cm^−1^ and C—H at 2923 cm^−1^ and 2850 cm^−1^, asymmetric stretching vibrations of C—NH—C at 1126 cm^−1^, bending vibrations of N—H at 1570 cm^−1^, and the vibration absorption band of C=O at 1635 cm^−1^, which was formed during the crosslink reaction between MA and EDA, forming the crosslinked polymer network structure.^33^ From 1 H NMR spectrum (Figure S2, Supporting Information), no characteristic peaks of aromatic structure appeared, which demonstrated that the fluorescence was not from extended conjugation structure but some special groups, namely sub‐fluorophores. Furthermore, the zeta potential of the SSFPCDs dilute aqueous solution (0.1 mg mL^−1^) was measured and the potential of the dilute solution was negative (−8.15 mV), which indicated that there were many carboxyl groups remaining on the surface. According to the thermogravimetric analysis (TGA), the SSFPCDs showed good thermal stability. The decomposition temperature of SSFPCDs was 220 °C, and the origin weight can still remain 90% by 300 °C (Figure S1f, Supporting Information).

The UV/Vis absorption spectrum of SSFPCDs aqueous solution exhibited two absorption peaks, focused at 344 and 425 nm (Figure 1c). Under UV excitation (365 nm), the dilute solution of SSFPCDs (1 mg mL^−1^) displayed strong blue fluorescence (460 nm) with the fluorescence QY of 18.9%, and exhibited excitation dependence, indicating several excited states existing in SSFPCDs.^38^, ^47^ Because the as‐prepared SSFPCDs have undergone a strict purification process, there is no extra small molecules contaminating the dots, as a result, the fluorescence is really originated from the SSFPCDs.

Besides the basic analysis and characterization above, some novel findings were exhibited to investigate the emission center of PCDs. First, excited by UV light (365 nm), the SSFPCDs powder showed strong red SSF (625 nm) unlike other reported PCDs, which do not have luminescence in the solid state because of the aggregation‐caused luminescence quenching (Figure 1d). The SSFPCDs powder also showed excitation dependent emission, which indicated several energy levels in solid state (Figure S3, Supporting Information). Second, the maximum emission (625 nm) of the powder possessed remarkable red‐shift of 165 nm than that of dilute aqueous solution (460 nm), which was larger than the shift between aggregate state and the solution state of organic molecules. Moreover, SSFPCDs exhibited tunable photoluminescence under the UV excitation according to the concentration aqueous dispersions. Fine‐tuning of the fluorescence emission wavelength across the entire visible spectrum, which were 460, 510, 575, and 625 nm, can be easily achieved by varying the concentration of the SSFPCDs dispersions (Figure 1e,f). To be specific, with a low solute concentration which was from 0.1 to 5 mg mL^−1^, SSFPCDs had only one emission peak on 460 nm. Then the emission peak reached 510 nm with the concentration of 6.5 mg mL^−1^ and when we further increased the solute concentration, the emission spectra showed another peak on 575 nm. The emission intensity ratio of 510–575 nm will decrease with the increase of solute concentration and finally only 575 nm emission left in the concentration of 16.5 mg mL^−1^ (**Figure** **2**a).

**Figure 2:**
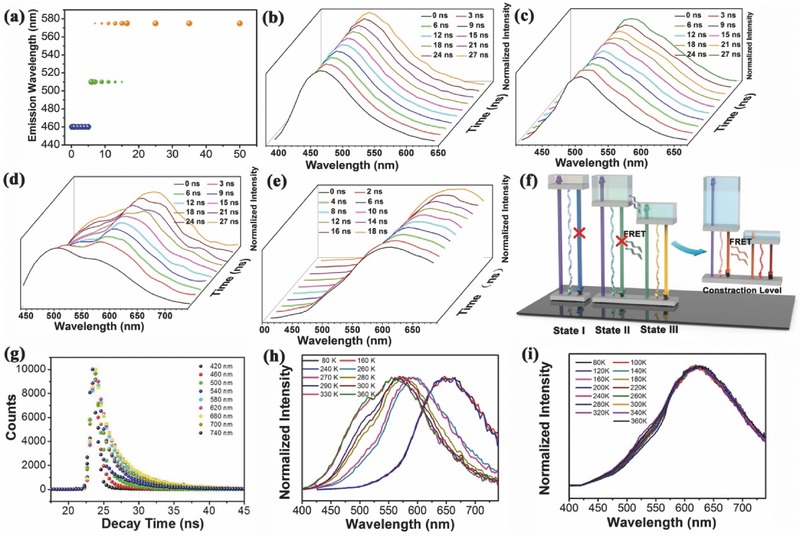
Photophysical properties of PCDs with 365 nm excitation light. a) The PL emission peak shift of SSFPCDs in aqueous solutions with concentration from 0.1 to 50 mg mL^−1^. Peak normalized TRES of SSFPCDs aqueous solution with concentration of b) 0.8, c) 6.5, and d) 16.5 mg mL^−1^ with the decay time on 0, 3, 6, 9, 12, 15, 18, 21, 24, and 27 ns at room temperature. e) Peak normalized TRES of SSFPCDs powder with the decay time on 0, 2, 4, 6, 8, 10, 12, 14, 16, and 18 ns at room temperature. f) Energy levels and fluorescent mechanism of SSFPCDs aqueous solutions and SSFPCDs powder. g) Fluorescence lifetimes of the 16.5 mg mL^−1^ SSFPCDs aqueous solutions at different wavelengths. h) Temperature‐dependent emission spectra of the 16.5 mg mL^−1^ SSFPCDs aqueous solutions. i) Temperature‐dependent emission spectra of the SSFPCDs powder.

The emission peak of PCDs was highly dependent on the concentrations. To reveal the mechanism of concentration‐dependent emission, time‐resolved emission spectra (TRES) of solutions with different concentrations were investigated. For peak normalized TRES of the solution with the concentration of 0.8 and 6.5 mg mL^−1^ (Figure 2b,c), the emission spectra had the same wavelength (460 nm blue emission for 0.8 mg mL^−1^ PCDs and 510 nm green emission PCDs for 6.5 mg mL^−1^ PCDs) during all the decay time from several to tens of ns, indicating that there was only one emission center in these two cases without any energy transfer process.^48^, ^49^ However, the solution with the concentration of 16.5 mg mL^−1^ had the time‐dependent spectra with the properties of a two‐state model (Figure 2d): the green emission at 510 nm appeared in the first several ns decay, while the yellow emission at 575 nm emerged during longer decay time. The two emission centers in 16.5 mg mL^−1^ PCDs suggested the energy transfer process between two excitation states.^48^, ^50^, ^51^, ^52^ Based on the above data, the emission model of SSFPCDs solution was schemed in Figure 2f. It was reasonable to attribute the extra‐large red‐shift to three fluorescence centers existing in SSFPCDs, including States I, II, and III, which were formed due to the fact that the plentiful sub‐fluorophores, formed through the crosslink process, were in different chemical environments. To be more specific, State I was originated from sub‐fluorophores existing on the surface, which was far away from State II and State III which were derived from close sub‐fluorophores protected by the polymer net. As a result, State II (the green emission band gap) and State III (the yellow emission one) had energy transfer process, while the State I (blue emission) was distinct from them. For SSFPCDs dilute solution possessing blue fluorescence, the State I was the dominant fluorescence center for the radiative recombination of electron–hole (e–h) pairs. However, without the protection from the network structure, fluorescence center from the State I will be quenched in high solute concentration thus facilitating the State II dominating the fluorescence. With further elevated concentration, there was energy transfer process from State II to State III, leading State III dominant while State II quenching.^53^, ^54^

Moreover, the fluorescence time‐correlated single‐photon counting (TCSPC) was also used to study the exciton behavior of SSFPCDs solutions with different concentrations. The average lifetimes of solution with the concentration of 0.8, 6.5, and 16.5 mg mL^−1^ were 8.74, 8.91, and 11.28 ns, respectively. On one hand, since State I and State II are independent emission centers, they display similar average lifetimes. On the other hand, the average lifetime of 16.5 mg mL^−1^ PCDs was increased from 8.91 to 11.28 ns, due to the energy transfer process from State II to State III.

The most important PL phenomenon of as‐prepared PCDs was the quench‐resistant red emission. In order to uncover the principle of red solid state fluorescence, TRES of solid powder was also investigated. The solid powder had the time‐dependent spectra with the properties of a continuous spectral shift (Figure 2e), indicating the existence of multiple continuous narrow band gaps.^50^, ^55^, ^56^, ^57^ Energy transfer process in the aggregated powder state can also be attested through the fluorescence TCSPC. The PL decay curves and the fitting results of the SSFPCDs, probed at different emission wavelengths with excitation at 365 nm, were shown in Figure 2g and Table S2 (Supporting Information). The average lifetimes of SSFPCDs powder vary from 0.6 to 4.02 ns with the probed emission wavelength from 420 to 740 nm. This is due to the fact that the energy transfer process competes with the radiative transition and nonradiative transition. As a result, the average lifetime increased and red‐shift emission is achieved.^53^, ^54^

According to the analysis above, the emission model of SSFPCDs powder was also schemed in Figure 2f. SSFPCDs, after aggregating to solid powder, contracted themselves, which is adequately supported by the size difference between aqueous state SSFPCDs and solid state SSFPCDs. The decreased distance between each sub‐fluorophore induced the electron distribution of SSFPCDs and thus leads to the contraction of previous band gaps as well as Förster resonance energy transfer (FRET) process.^58^, ^59^ Since the fluorescence state of SSFPCDs will be contracted into several narrower continuous band gaps with FRET process, an unusual red fluorescence is achieved. To further confirm this mechanism, the temperature‐dependent emissions of both the high concentration solution (16.5 mg mL^−1^) and the SSFPCDs powder were tested and analyzed. The high concentration solution possessed temperature‐dependent PL; at low temperatures below 273 K, the high concentration SSFPCDs solution had a red shift emission wavelength, which is quite similar with that of the powder state (Figure 2h) and high temperatures quenched the PL intensity to some degree (Figure S5, Supporting Information). The red‐shift was owing to the narrower band gap in solid state, which was similar to powder state. The PL quenched behavior can prove that the crosslinked sub‐fluorophores was the main radiative process path, since the high temperature increased the vibration and rotation, thus facilitating the nonradiative process.^7^ While for SSFPCDs in powder state, due to the fact that their phase state changes little, their emission wavelength was fixed with the changing temperature from 82 to 360 K (Figure 2i).

Furthermore, the unique self‐quenching resistant performance in solid state is mainly ascribed to the crosslinked polymer chains with net structure and the unique fluorescence centers of PCDs, which were named sub‐fluorophores. Since small molecular dyes exhibited fluorescence due to typical conjugated π‐domains fluorophores, their fluorescence suffered from π–π interaction, a major energy‐transfer process that may cause self‐quenching in solid state.^24^, ^25^ Unlike them, it is sub‐fluorophores in SSFPCDs that is the key of fluorescence, which do not undergo the π–π interaction process and thus prevent SSFPCDs with aggregation state from quenching in some extent. Moreover, due to the protection from the network structure of polymer chains, encapsulating the sub‐fluorophores, these sub‐fluorophores cannot directly attached to each other even in solid state, which was the main reason for quench‐resistant solid‐state fluorescence.

According to the above illustrated PL mechanism, we have successfully obtained powder with tunable SSF by adjusting the ratio of PCDs to starch. As a result SSFPCDs–starch composites with blue, green, and yellow SSF were achieved which were named as b‐PCDs, g‐PCDs, and y‐PCDs powders (**Figure** **3**), respectively, by dispersing the SSFPCDs solution in the starch matrix through an efficient method described in the Experimental Section. These SSFPCDs aqueous solutions with different concentrations can also be used as fluorescence inks with different fluorescence emissions from blue, green, to yellow, which can be written on paper and maintain the original fluorescence intensity even after one month storage at room temperature (Figure 3). In Figure 3 the fabrication of full‐color emission films is also shown. In this case, polyvinyl alcohol (PVA) played the role as matrix to help the SSFPCDs solutions with different concentrations maintain their fluorescence when processed in film form. Because PVA has the advantages of high transmission, good water solubility, and easy processing, the SSFPCDs/PVA films were transparent under sunlight and emit light from blue to red under UV light (365 nm).

**Figure 3:**
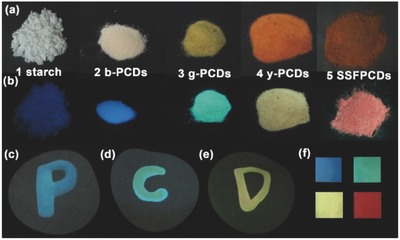
Nanocomposite and print ink applications of PCDs. Photographs of starch (No.1), b‐PCDs (No.2), g‐PCDs (No.3), y‐PCDs (No.4), and SSFPCDs (No.5) powders under a) daylight and b) UV light of 365 nm. c–e) Photographs of SSFPCDs fluorescence ink under UV light (365 nm). f) SSFPCDs/PVA film with full‐color emission under UV light (ex = 365 nm).

Beneficial due to their unique properties and performance, these fluorescence powders can be used in various forms to satisfy the requirements of different important applications. These fluorescent powders with different emissions can be utilized as the phosphor in LED application. The methods to prepare them are described in the Experimental Section.^60^ Solid LEDs with various color emissions were fabricated with the Commission Internationale de L'Eclairage 1931 (CIE) coordinates of (0.59, 0.41), (0.43, 0.48), (0.30, 0.49), and (0.26, 0.33), respectively. By combining the b‐PCDs powder and the SSFPCDs powder, a white lighting solid LED with the CIE coordinate approaching to (0.31, 0.31) has also been achieved (**Figure** **4**), realizing full‐wavelength emission LEDs with SSFPCDs of the same chemical structure.

**Figure 4:**
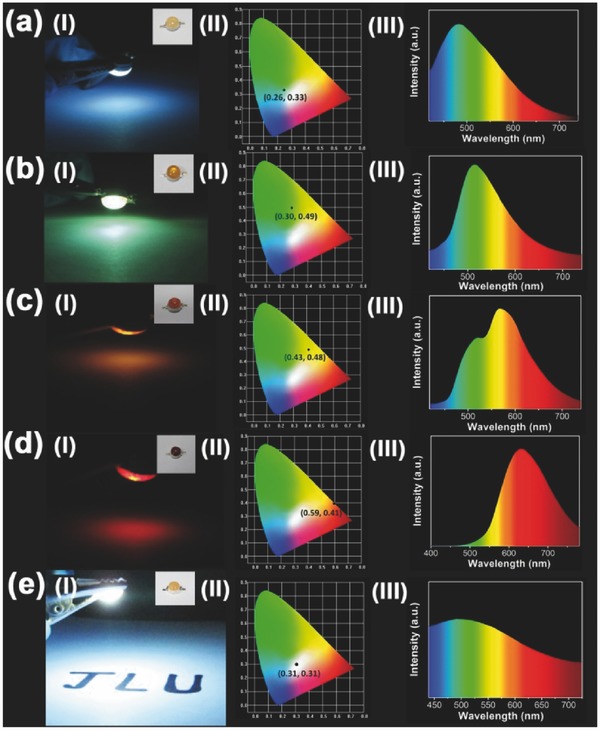
LED prototypes, prepared by mixing SSFPCDs composites or SSFPCDs powder and curable resin, with a) blue, b) green, c) yellow, d) red, and e) white light. (I) Fluorescent images, (II) PL emission spectra, and (III) CIE chromaticity coordinate of the LEDs. Inset: Optical images of the LEDs.

In summary, SSFPCDs with efficient full‐color SSF were realized through a one‐pot microwave‐assisted method. The polymer net structure, formed through the crosslink process, can prevent the emission centers of SSFPCDs from getting too close and the nonconjugated sub‐fluorophores help SSFPCDs avoid π–π interaction quenching, helping the SSFPCDs conquer self‐quenching in the aggregation state, which was a huge improvement in the field of SSF. Meanwhile, the existence of three fluorescence centers in SSFPCDs can lead to full‐color concentration‐dependent emission. Based on the interesting and efficient PL mechanism, SSFPCDs or SSFPCDs–starch composites with tunable SSF from red to blue have been realized. Furthermore, solid LEDs of various color emission from red to blue have been obtained. By combining b‐PCDs powder and SSFPCDs powder, a white light emitting LED has been achieved. Meanwhile, SSFPCDs/PVA film with full‐color emission under UV light (365 nm) can also be achieved, indicating that SSFPCDs can be fabricated in various forms to meet the requirements of different applications.

## Experimental Section

*Materials*: MA (99.9%), EDA (standard for GC, 99.5%), PVA (*M*_n_ = 77 000, 99% hydrolyzed), and starch from potato were purchased from Sigma‐Aldrich without further purification.

*Preparation of Polymer Carbon Dots*: The SSFPCDs were prepared by MA and EDA through microwave. In general, 2.3214 g of MA was dissolved in 20 mL deionized water to form a transparent solution; then 1340 µL EDA was added to the solution, which was then stirred for a while to form a homogeneous solution. The solution was then put into a microwave oven with the power of 1000 W for 20 min, during which the solution changed from light yellow liquid to orange solid powder, indicating the formation of SSFPCDs. Then these orange powders were dispersed in 40 mL deionized water, followed by centrifugation (9000 r min^−1^, 15 min) to remove large indissoluble particles. Finally, a 3500 dialysis bag was used for 3 d to further purify the sample and a stable brown powder was collected by freeze‐drying.

*Preparation of Starch/SSFPCDs Composites (b‐PCDs, g‐PCDs, and y‐PCDs Powder)*: The starch/SSFPCDs composites (mass ratio = 1:2, 4:1, and 25:1), which show fluorescence of yellow, green, and blue, respectively, were synthesized by a simple method. Mixing the SSFPCDs and starch in different ratio in water and stirring for 48 h, the raw products were obtained. Then the reaction mixtures were filtered to get rid of unabsorbed SSFPCDs, while the remaining solid product on filter paper were put in an oven under 30 °C to remove excess water and make them dryer. Finally, the starch/SSFPCDs composite were prepared by grinding the dried powder in agate mortar and screening by mesh sieve. The fluorescence was controlled by modulating the starting ratio of starch to SSFPCDs, which will affect the coverage degree of SSFPCDs on starch particles and then changed the fluorescence of starch/SSFPCDs composite. These composites were kept in a vacuum oven and used as phosphors in further experiments.

*Fabrication of LEDs Based on SSFPCDs and Starch/SSFPCDs Composites*: Commercially available GaN LED chips without phosphor coating were purchased from Advanced Optoelectronic Technology Inc. The emission of the GaN LED chips centered at 365 nm, and the operating voltage was 4.0 V. In the preparation of the color conversion layer, SSFPCDs or starch/SSFPCDs composites were meshed to a fine powder and then mixed with the curable resin according to our previous method. Finally, they were put into a vacuum chamber to remove bubbles. After that, the mixtures were used to fill the cup‐shaped void of an LED chip. After being immobilized under UV light for 5 min, the LEDs from SSFPCDs or starch/SSFPCDs composites were fabricated.

*Fabrication of the White Emission LEDs*: The blue emission starch/SSFPCDs composites (25:1) were mixed with SSFPCDs powder at the mass ratio of 8:1. After meshing the mixtures into fine powder and mixed them with the curable resin. The white emission LEDs were obtained in the above method.

*Preparation of SSFPCDs/PVA Film with Full‐Color Emission*: The SSFPCDs/PVA films which show fluorescence of red, yellow, green, and blue, respectively, were synthesized by a simple method. PVA solutions with the concentration of 5% were prepared by dispersing 5 g into 95 g deionized water under 100 °C. Then in order to form a uniform state, 10, 125, and 500 mg SSFPCDs powder were added into the 5% PVA solution of 10 mL under sonication, respectively. After molding on a piece of smooth glass in room temperature for 48 h, SSFPCDs/PVA films with blue, green, and yellow fluorescence can be prepared. The PVA solution with concentration of 12% is prepared in the same way. 5 g SSFPCDs powder were added into the 12% PVA solution of 10 mL under sonication. After molding in the same way, SSFPCDs/PVA films with red fluorescence can be achieved.

*Characterization*: High‐resolution transmission electron microscopy was recorded on JEM‐2100F and FEI Tecnai F20 (the decreased electrobeam intensity and increased exposure time can be beneficial to the obtained TEM images of PCDs). AFM images were recorded in the tapping mode with a Nanoscope IIIa scanning probe microscope from Digital Instruments under ambient conditions. Fluorescence spectroscopy was performed with a Shimadzu RF‐5301 PC spectrophotometer. UV–vis absorption spectra were obtained using a Shimadzu 3100 UV–vis spectrophotometer. IR spectra were taken on a Nicolet AVATAR 360 FT‐IR spectrophotometer. XPS was investigated by using ESCALAB 250 spectrometer with a mono X‐Ray source Al Kα excitation (1486.6 eV). Binding energy calibration was based on C 1s at 284.5 eV, N 1s at 285.5 eV, and O 1s at 288.0 eV. Elemental analysis was performed on Elementar Vario MICRO CUBE, all data were parallel at least twice and the average values were obtained by measuring three kinds of sample batches. DLS measurements and Zeta potential were measured using the Zetasizer Nano‐ZS (Malvern Instruments). The sample was measured five times and the average data were presented. The absolute quantum yield was measured on a fluorescence life‐time and steady‐state spectrometer (Edinburgh Instrument, FLS 920, with an integrating sphere). NMR spectra were performed with a Bruker AVANCE NMR spectrometer (500 MHz) using D_2_O as the solvent. TGA was measured on a Mettler Toledo TGA/SDTA851e instrument under N_2_ atmosphere from room temperature to 600 °C with a heating rate of 10 °C min^−1^. The color of the light was identified by the CIE calorimeter system.

*Time Resolved Photoluminescence*: Nanosecond fluorescence lifetime experiments were performed by the TCSPC system under right‐angle sample geometry. A 379 nm picosecond diode laser (Edinburgh Instruments EPL375, repetition rate 2 MHz) was used to excite the samples. The fluorescence was collected by a photomultiplier tube (Hamamatsu H5783p) connected to a TCSPC board (Becker&Hickel SPC‐130). Time constant of the instrument response function was about 300 ps.

## Conflict of Interest

The authors declare no conflict of interest.

## Supporting information

Supplementary
