# Supplementary material for: Full‐Color Emission Polymer Carbon Dots with Quench‐Resistant Solid‐State Fluorescence
Source: Adv Sci (Weinh). 2017 Sep 28;4(12):1700395. doi: 10.1002/advs.201700395 (PMC5737236; doi:10.1002/advs.201700395)
Supplement: Supplementary file 1 — Supplementary [file ADVS-4-na-s001.pdf]

Copyright WILEY-VCH Verlag GmbH & Co. KGaA, 69469 Weinheim, Germany, 2017.

ADVANCED SCIENCE
Open Access

Supporting Information

for *Adv. Sci.,* DOI: 10.1002/advs.201700395

Full-Color Emission Polymer Carbon Dots with Quench-Resistant Solid-State Fluorescence

*Jieren Shao, Shoujun Zhu, Huiwen Liu, Yubin Song, Songyuan Tao, and Bai Yang**

Supporting Information

Full-Color Emission Polymer Carbon Dots with Quench-Resistant Solid-State Fluorescence

*Jieren Shao, Shoujun Zhu, Huiwen Liu, Yubin Song, Songyuan Tao, Bai Yang**

J. R. Shao, Dr. S. J. Zhu, H. W. Liu, Y. B. Song, S. Y. Tao, Prof. B. Yang

State Key Laboratory of Supramolecular Structure and Materials, College of Chemistry, Jilin University, Changchun, 130012, P. R. China.

E-mail: byangchem@jlu.edu.cn

Dr. S. J. Zhu

Department of Chemistry, Stanford University, Stanford, California 94305 (USA).

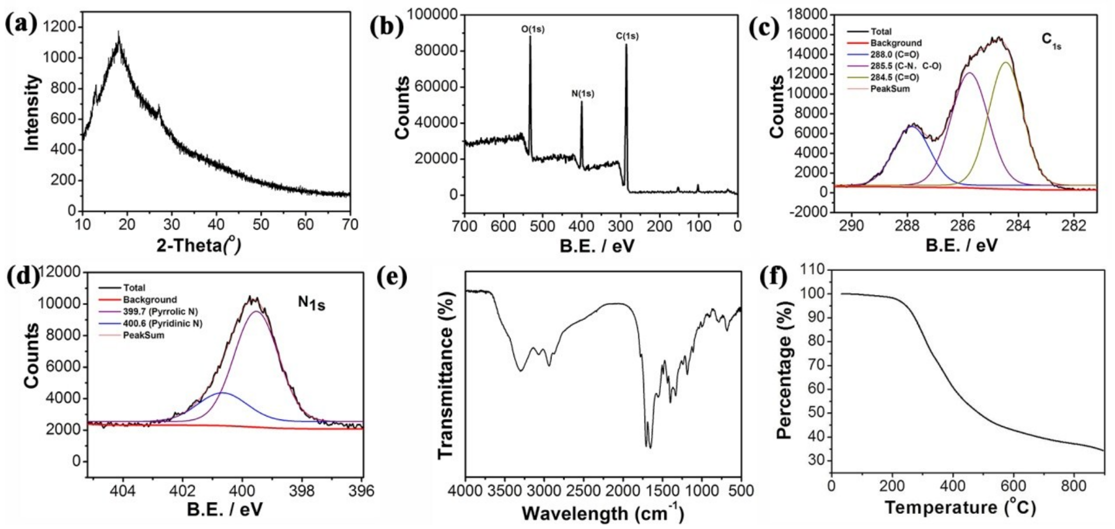

Figure S1. a) XRD pattern, b) XPS survey spectrum, c) High resolution scans of C 1s, d) high resolution scans of N 1s, e) FT-IR spectrum, f) TGA spectrum of the SSFPCDs.

H (%)	C (%)	N (%)	O (%)
6.28	48.25	18.48	26.99

Table S1. Element analysis of SSFPCDs.

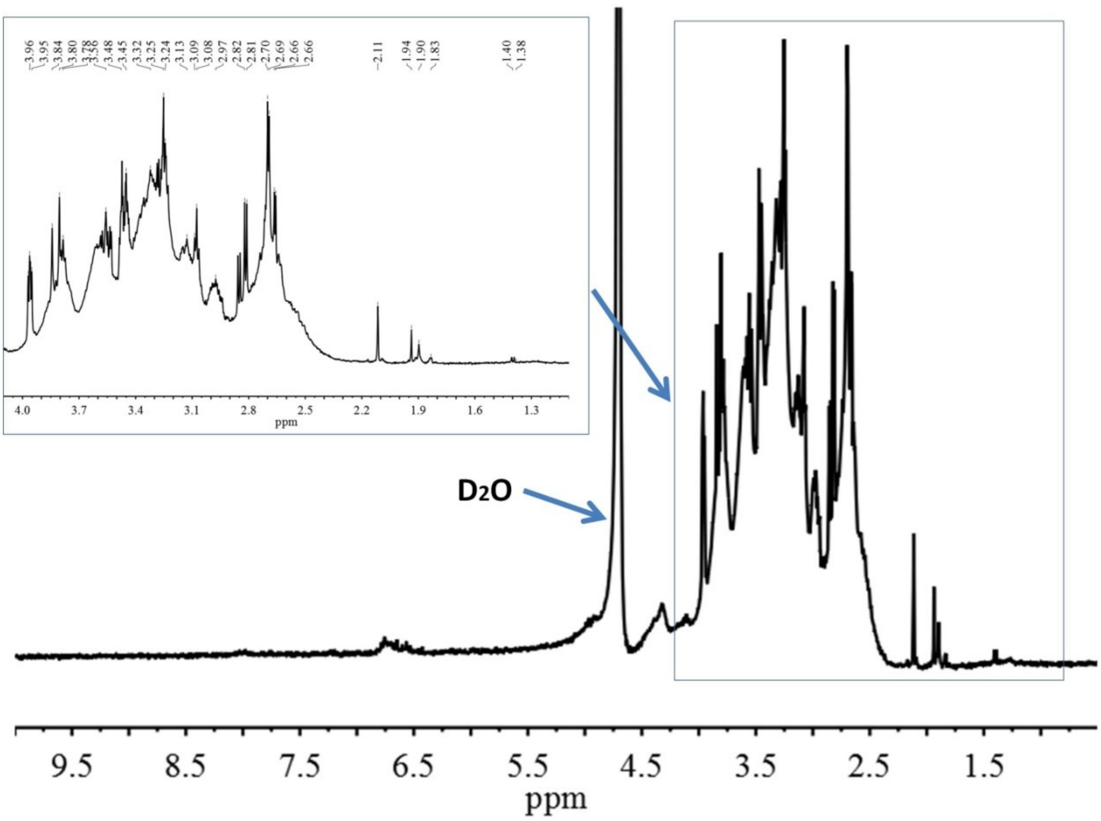

Figure S2. The 1H NMR spectrum of the SSFPCDs in D_2O.

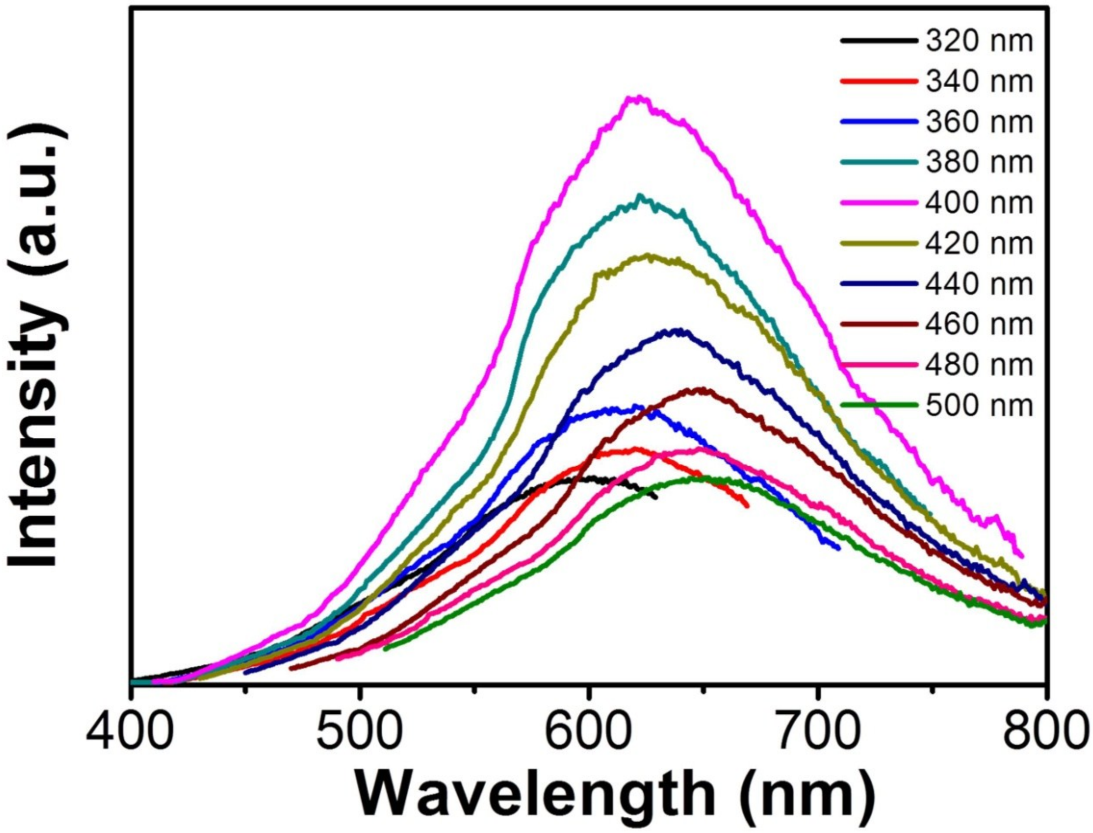

Figure S3. PL emission spectrum of the SSFPCDs powder.

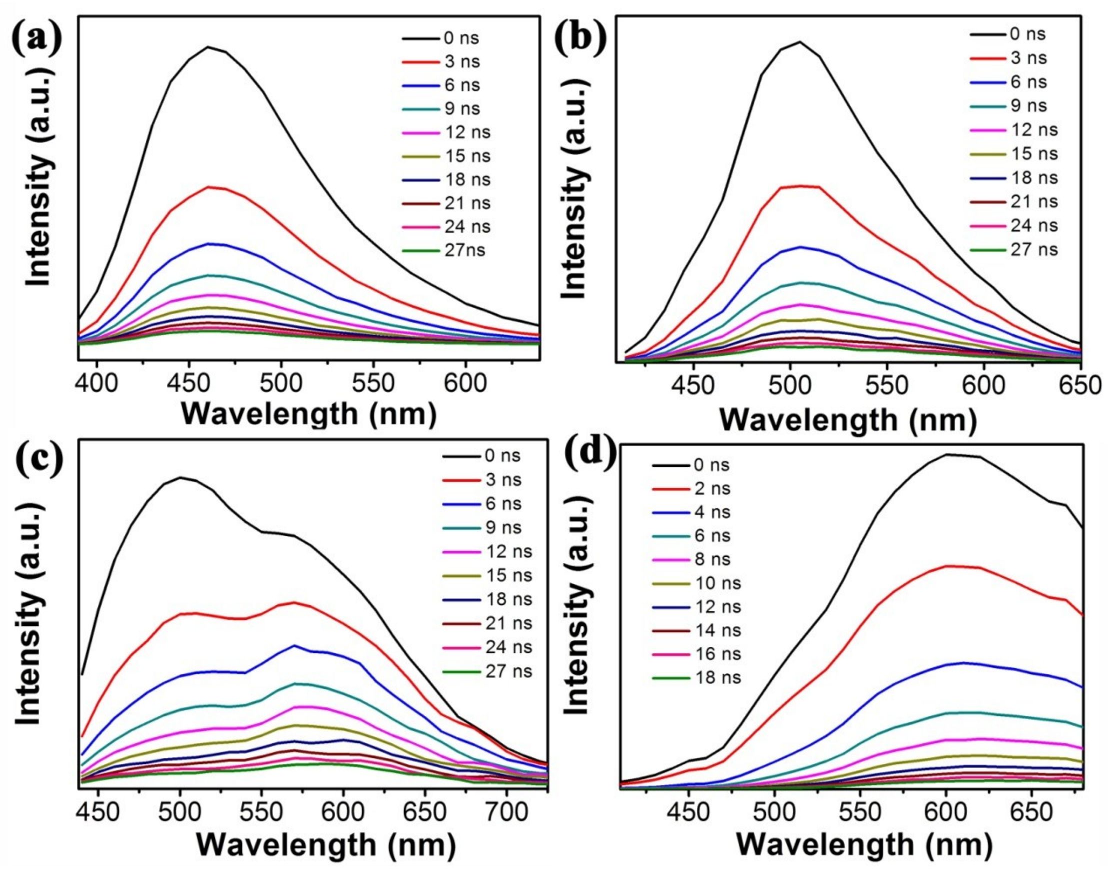

Figure S4. 365 nm excitation is adopted. TRES of SSFPCDs aqueous solution with concentration of a) 0.8 mg/mL, b) 6.5 mg/mL, and c) 16.5 mg/mL with the decay time on 0, 3, 6, 9, 12, 15, 18, 21, 24, and 27 ns at room temperature. d) TRES of SSFPCDs powder with the decay time on 0, 2, 4, 6, 8, 10, 12, 14, 16, and 18 ns at room temperature.

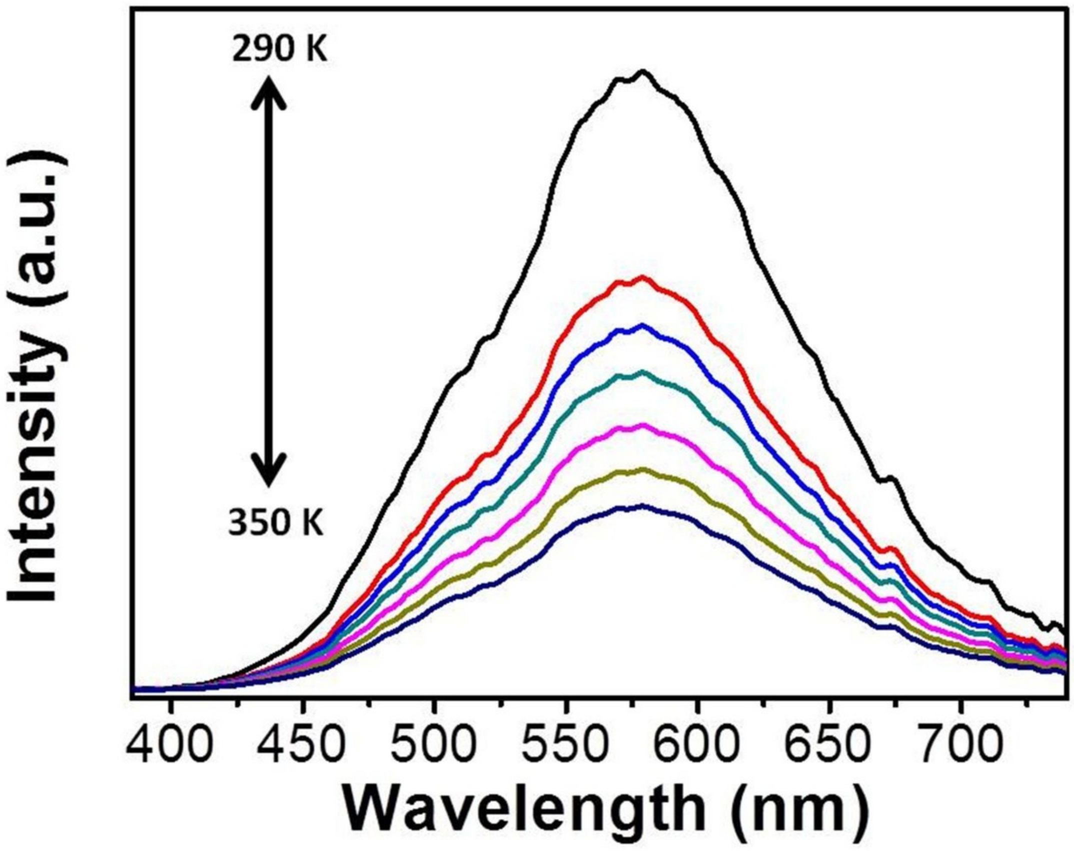

Figure S5. The temperature dependent PL of SSFPCDs solution with the concentration of 16.5 mg/mL (ex=365 nm).

□λ_{em}/nm	τ_1/ns	B_1/%	τ_2/ns	B_2/%	avg/ns	CHISQ
420	0.43	93.43	3.08	6.57	0.60	1.730
460	0.67	78.78	2.66	21.22	1.09	1.508
500	0.90	65.74	3.26	34.26	1.71	1.169
540	1.37	52.90	4.61	47.10	2.90	1.202
580	1.91	53.16	5.47	46.84	3.58	1.289
620	2.10	50.68	5.75	49.32	3.90	1.259
660	2.30	53.63	6.01	46.37	4.02	1.110
700	1.37	38.76	5.08	61.24	3.64	1.434
740	0.71	32.98	4.43	67.02	3.20	1.473

Table S2. Fluorescent lifetimes of the 16.5 mg/mL SSFPCDs aqueous solution at different wavelengths.
